# Mediating Effect of Work Stress on the Associations Between Psychological Job Demands, Social Approval, and Workplace Violence Among Health Care Workers in Sichuan Province of China

**DOI:** 10.3389/fpubh.2021.743626

**Published:** 2021-10-27

**Authors:** Xiaxia Sun, Mutian Qiao, Jianjun Deng, Juying Zhang, Jingping Pan, Xueli Zhang, Danping Liu

**Affiliations:** ^1^Department of Infection Control, West China University Second Hospital, Sichuan University, Chengdu, China; ^2^Key Laboratory of Birth Defects and Related Diseases of Women and Children, Sichuan University, Ministry of Education, Chengdu, China; ^3^Department of Health-Related Behavior and Social Medicine, West China School of Public Health and West China Fourth Hospital, Sichuan University, Chengdu, China; ^4^Department of Paediatrics, Western Women's and Children's Research Institute, West China University Second Hospital, Sichuan University, Chengdu, China; ^5^Department of Pediatrics, Maternal and Child Health Hospital of Zigong, Zigong, China; ^6^Department of Epidemiology and Biostatistics, West China School of Public Health and West China Fourth Hospital, Sichuan University, Chengdu, China; ^7^Health Information Centre of Sichuan Province, Chengdu, China

**Keywords:** health care workers, workplace violence, work stress, psychological job demands, social approval

## Abstract

**Objective:** The aim of this study was to investigate the prevalence of workplace violence against health care workers, to explore the combined association of work stress, psychological job demands, and social approval with workplace violence and their respective mechanisms among health care workers.

**Methods:** Using data from the Chinese Sixth National Health Service Survey (NHSS) in 2018 conducted among 1,371 health care workers in Sichuan province of China. A self-administered structured questionnaire was used to collect data on health care workers' socio-demographic and work-related characteristics, work stress, psychological job demands, social approval, and workplace violence. We used structural equation modeling (SEM) to test the hypothesized relationship among the variables.

**Results:** The results showed that a total of 77.0% health care workers were exposed to workplace violence. Work stress was directly related to workplace violence (β = 2.167, 95%CI: 1.707, 2.627), while psychological job demands and social approval had indirect associations with workplace violence *via* work stress [β = 0.427, 95%CI: 0.297, 0.557; β = −0.787, 95%CI: (−0.941)–(−0.633)]. Both psychological job demands (β = 0.197, 95%CI: 0.139, 0.255) and social approval [β = −0.346, 95%CI: (−0.399)–(−0.294)] had direct associations with work stress, while social approval had direct association with psychological job demands [β = −0.085, 95%CI: (−0.136)–(−0.034)]. Psychological job demands mediated the relationship between social approval and work stress.

**Conclusion:** Overall, decreasing workplace violence among health care workers requires to promote interventions to reduce work stress and psychological job demands by improving social approval.

## Introduction

Workplace violence is defined as violent events that could invoke implicit or explicit challenge to staff safety, well-being, or health through abusive, threatening, or assaulting behaviors and emotions in their working workplace (1). Such violence ranges from physical violence to psychological violence (2). Physical violence is the use of physical force, such as beating, kicking, slapping, stabbing, shooting, pushing, biting and pinching against another person or group. Psychological violence, on the contrary, includes verbal abuse, bullying/mobbing, harassment and threatening against another person or group. Workplace violence has long been acknowledged as a global problem, particularly among health care workers, the professionals who are most vulnerable to workplace violence (3). Workplace violence from patient and visitor is a primary occupational hazard for health care workers (4).

Numerous studies conducted in different countries have reported high levels of workplace violence among health care workers [e.g., Australia (5), New Zealand (6), and the United Kingdom (7)], and there is a general belief that it is increasing, same in China (8, 9). Exposure to workplace violence may affect health of health care workers (10). Health care workers who were exposed to workplace violence had a lower quality of life than those who were not exposed to workplace violence (11). High anxiety, depressive symptoms and sleep disturbances were also associated with frequent workplace violence (12, 13). In addition, workplace violence significantly affects health care workers' job satisfaction and work engagement (14), declines work enthusiasm and work efficiency, thus leads to increased job burnout and turnover intention (15). Furthermore, workplace violence in the health sector, particularly in developing countries, seriously undermines health service environment (16), the quality of health services, the retention of health professionals and the effectiveness of health care systems (17).

As a result, an increasing amount of studies have been conducted on health care workers to explore risk factors for workplace violence. Traditionally, both patient characteristics (such as having a severe mental disorder), and socio-demographic characteristics of health care workers [i.e., age, gender, education level (18, 19)] predict workplace violence. In addition, work-related characteristics [i.e., profession, department, hospital type, professional title, work in shifts, years of work experience, and previous workplace violence training (20–22)] are considered as influencing factors of workplace violence. In addition, problems in psychosocial work environments may also contribute to the occurrence of patients' aggression (12, 23, 24), including work-related social support, work stress, psychological job demands, perception of the practice environment, etc.

Work stress usually refers to physical and mental health pressures, and body function disorders, due to the imbalance between staff's ability and their objective demands (25). Studies have shown that work stress significantly predicts negative outcomes, such as patient aggression (26, 27). Balducci et al. showed in their study that health care workers' experience of stress at work may make them more vulnerable to workplace violence (24). In a longitudinal study, Magnavita indicated that workers with work strain at baseline had a significant risk of being subject to aggression in the following year (12).

Psychological job demands refer to aspects of a job that require sustained psychological effort (28). It is a combination of stressors such as work load, unexpected tasks, and job-related interpersonal conflict and have mainly been operationalized in terms of work amount combined with time pressure (29). Job demands (including psychological and physical job demands) were identified as significant predictors of workplace violent threat. High levels of job demands were associated with more patient aggression among health care workers (12). In addition, as one of the sources of work stress, high levels of job demands also increase work stress (29, 30).

Social approval refers to workers' perception of the practice environment, from the perspective of health care workers, it mainly includes the perception of public trust, recognition and respect for their job, doctor-patient relationship, etc (31). Practice environment was demonstrated to be associated with workplace violence against health care workers. Previous study has showed that nurses who worked in poor practicing environment had greater odds of experiencing violence (32). The literature has shown that lack of trust and respect in the workplace are two antecedents of workplace conflict among nurses (33). Trenoweth found that the development of nurse-patient relationship is a protective factor against violence risk (34). Moreover, supportive practice environment, as a kind of job resource, can also increase work engagement and reduce the time required, thus decreasing psychological job demands through the motivation process (35). In addition, poor practice environment [like unsatisfactory doctor-patient relationship (36), lack of respect by the community (37)] also increase work stress among health care workers.

**Figure 1:**
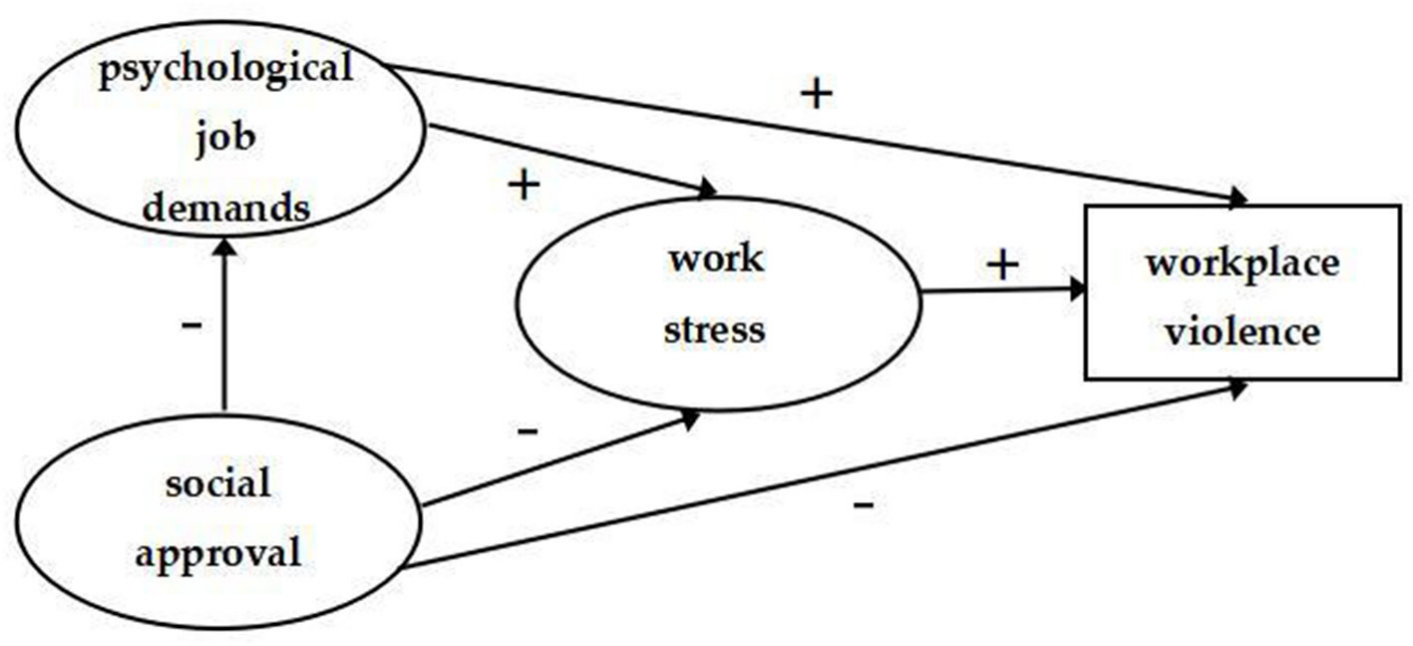
The theoretical model and hypotheses.

Previous studies have examined the relationships between one of the above psychosocial work environments factors and workplace violence among health care workers, but comparatively little is known about the combined effects of these three factors or the underlying mechanisms of the relationships. Based on the above, we examined the relationships among work stress, psychological job demands, social approval, and workplace violence in health care workers in Sichuan province of China. The hypothesized model is shown in Figure 1. Specifically, work stress has a direct positive effect on workplace violence (hypothesis 1), social approval has direct negative effect on workplace violence (hypothesis 2), work stress (hypothesis 3), and psychological job demands (hypothesis 4). We also hypothesize that psychological job demands have direct positive effect on both workplace violence (hypothesis 5) and work stress (hypothesis 6). In addition, we suggest that the relationship between social approval and workplace violence is mediated by work stress (hypothesis 7), the relationship between psychological job demands and workplace violence is mediated by work stress (hypothesis 8) and the relationship between social approval and work stress is mediated by psychological job demands (hypothesis 9).

This study is the first to explore the combined association of work stress, psychological job demands, and social approval with workplace violence and their respective mechanisms among health care workers in China. Findings from this study may provide important references for strategies to decrease workplace violence and improve medical service environment, promote health care workers' job satisfaction and health, increase effectiveness and quality of medical care.

## Materials and Methods

### Settings and Participants

This research used data from the Chinese sixth National Health Service Survey (NHSS) in 2018, conducted among health care workers in Sichuan province, China. All health care workers who had a practicing qualified certificate on file in the tertiary hospitals, secondary hospitals, community health centers, and township hospitals of Sichuan Province were eligible to be study subjects.

Multistage stratified random sampling was used to acquire the study sample. In the first stage, 14 cities were randomly selected from 21 prefecture-level cities. In the second stage, all the third-class comprehensive hospitals and some of the second-class hospitals were selected in the 14 cities, and a total of 70 towns and communities were randomly selected in these cities. All the community health service centers or township hospitals in the 70 towns or communities were enrolled in the investigated medical institutions. In the third stage, a total of 20 clinical physicians and 10 nurses were selected from each third-class comprehensive and second-class hospital by simple random sampling. Five physicians, three nurses, and two public health workers were randomly selected from each community health service center and township hospital.

The questionnaire was self-administered. Informed consent was obtained from each health care worker following a detailed explanation about the purpose of the study. A total of 1,685 health care workers were eligible to participate in 2018. We excluded 314 questionnaires from analysis because information was incomplete, with 1,371 questionnaires valid in this analysis (effective response rate: 81.4%).

### Ethical Consideration

The study protocol was approved by the ethics committee of the National Health Commission of the People's Republic of China. Verbal consent was obtained from each participant following a detail explanation about the purpose of the study.

### Measures

Respondents' socio-demographic and work-related characteristics, perceived work stress, psychological job demands, social approval, and exposure to workplace violence were collected using questionnaires.

#### Socio-Demographic and Work-Related Characteristics

Socio-demographic characteristics included age, gender, marital status, education level, individual annual income. Work-related characteristics included practice setting, profession, professional title, management responsibility, working in shifts, and hours worked per week.

#### Work Stress

The Chinese version of the Effort-Reward Imbalance (ERI) model was used to measure work stress (38). The ERI model is suitable for research on work stress in health professions (39) and consists of three dimensions: extrinsic efforts, rewards, and overcommitment. The items are scored from 1 to 4, with higher scores indicating higher demands of efforts, overcommitment, and rewards. Effort-reward ratio (ERR) was calculated using a predefined algorithm that quantifies the degree of mismatch between high cost and low gain. The ERI ratio (ERR) is given by [effort score/(reward score ^*^ k)], where k is a correction factor. The correction factor is the ratio of the number of items of effort and rewards used to adjust for unequal items on the subscales (3/7 = 0.4286 in this study). An ERR of >1 reflects a perception of an imbalance between efforts and rewards. In this study, we used ERR and overcommitment as observation variables to measure work stress. In this study, the Cronbach's Alpha coefficients of the scale was 0.736.

#### Psychological Job Demands

Psychological job demands were assessed with three items derived from the Chinese version of Job Content Questionnaire (C-JCQ) (40): (1) My work requires a high level of skill, (2) My work requires me to take on a lot of responsibilities, (3) My work requires long periods of intense concentration on the task. Four-point Likert scale ranging from 1 (highly disagree) to 4 (highly agree) was utilized to evaluate all these items, a higher score indicated higher identification of psychological job demands. The Cronbach's Alpha coefficients of this scale in this study was 0.774.

#### Social Approval

Social approval was assessed by a five-item survey asking participants to answer five questions about the practice environment they perceived, including patients' trust, respect, and recognition to health care workers, the public respect for them and patient-doctor relationship. Items are rated on a five-point Likert scale from 1 (very low or poor) to 5 (very high or good). The total score was calculated by adding the response score for each item and ranged from 5 to 25 (5–11 = low; 12–18 = moderate; 19–25 = high) with higher scores indicating higher social approval. In the current study, Cronbach's alpha of the scale was 0.814.

#### Exposure to Workplace Violence

The exposure status of workplace violence was categorized into two basic types: 0 for no and 1 for yes. Workplace violence was defined in this study as any incident where health care workers experiences any of the following: (1) verbal abuse, (2) physical abuse, and (3) emotional abuse (such as hurtful attitudes or remarks). Health care workers were asked to indicate if they had experienced any of the three types of violence within the past 6 months.

### Statistics Analysis

Data were entered using the Epidata 3.1 database and were analyzed using the IBM SPSS version 23.0 (SPSS Inc., Chicago, IL, USA) and Mplus 7.11 (Muthén & Muthén, Los Angeles, CA, USA).

We first used descriptive statistics to examine socio-demographic and work-related characteristics of participants, and workplace violence status. Second, we undertook a descriptive analysis of respondents' work stress, psychological job demands and social approval, using means and standard deviations (SD). Third, a structural equation model (SEM) was employed to further test the hypothesized relationships among work stress, psychological job demands, social approval, and exposure status of workplace violence of respondents.

We performed SEM using the maximum likelihood estimation method to test the hypotheses (41). We used the subscale score of work stress, psychological job demands and social approval, as measurement variables and the total scores of these measures as latent variables. The binary variable exposure status of workplace violence was also included as a measurement variable. To examine whether the estimated model fit the data, we employed 4-fit indices with their respective cutoffs (42, 43): root mean square error of approximation (RMSEA) < 0.08; Tucker–Lewis index (TLI) and comparative fit index (CFI) values > 0.90; and a χ^2^/df of <5. If all indices demonstrate values close to or higher than these cutoff values, the model is considered to have a good fit to the data. Statistical significance was set at *p* < 0.05.

## Results

### Socio-Demographic and Work-Related Characteristics of Respondents

Table 1 shows the socio-demographic and work-related characteristics of 1,371 respondents. Overall, 1,055 (77.0%) of the respondents experienced workplace violence. The average age of health care workers was 36.9 ± 10.0 years. Over half of the respondents were female (63.8%). Most of the respondents were married (78.8%). 49.9% of the respondents were with associate's degree and below. 47.6% of the respondents had an individual annual income of $7,500–14,999.

**Table 1:** Socio-demographic and work-related characteristics of respondents (*n* = 1,371).

**Characteristics**	**Total** ***n***	**Without** **workplace violence**	**With** **workplace violence**	**χ^2^**	***P*** **-value**
**Socio-demographic Characteristics**					
Gender				0.333	0.564
Female	875	206 (23.5)	669 (76.5)		
Male	496	110 (22.2)	386 (77.8)		
Age				1.237	0.539
18–29	366	81 (22.1)	285 (77.9)		
30–44	681	153 (22.5)	528 (77.5)		
≥45	324	82 (25.3)	242 (74.7)		
Marital status				0.033	0.856
Currently single^*^	290	68 (23.4)	222 (76.6)		
Married	1081	248 (22.9)	833 (77.1)		
Education level				1.911	0.385
Associate's degree and below	684	167 (24.4)	517 (75.6)		
Bachelor's degree	593	126 (21.2)	467 (78.8)		
Master's degree and above	94	23 (24.5)	71 (75.5)		
Individual annual income, $				4.887	0.087
<7,500	578	150 (26.0)	428 (74.0)		
7,500–14,999	653	135 (20.7)	518 (79.3)		
≥15,000	140	31 (22.1)	109 (77.9)		
**Work-related Characteristics**					
Practice setting				10.664	0.001
Community health centers and township hospitals	526	146 (27.8)	380 (72.2)		
Secondary or tertiary hospitals	845	170 (20.1)	675 (79.9)		
Profession				16.855	<0.001
Physician	795	179 (22.5)	616 (77.5)		
Nurse	425	83 (19.5)	342 (80.5)		
Public health workers	151	54 (35.8)	97 (64.2)		
Professional title				6.824	0.078
No	72	23 (31.9)	49 (68.1)		
Junior	716	172 (24.0)	544 (76.0)		
Intermediate	381	85 (22.3)	296 (77.7)		
Senior	202	36 (17.8)	166 (82.2)		
Has management responsibility				0.407	0.523
No	1049	246 (23.5)	803 (76.5)		
Yes	322	70 (21.7)	252 (78.3)		
Working in shifts				27.927	<0.001
No	413	133 (32.2)	280 (67.8)		
Yes	958	183 (19.1)	775 (80.9)		
Hours worked per week				16.717	<0.001
≤ 40	484	142 (29.3)	342 (70.7)		
41–48	224	43 (19.2)	181 (80.8)		
>48	663	131 (19.8)	532 (80.2)		

* *currently single includes single, divorced, and widowed*.

Over half of the participants practiced in secondary or tertiary hospitals (61.6%), were physicians (58.0%) and had a junior professional title (52.2%). Only 23.5% of the respondents had management responsibility. 16.3% of the respondents worked 41–48 h per week and 48.4% of the respondents worked more than 48 h per week. Differences are statistically significant in practice setting, profession, shift of work and hours worked per week between respondents with and without workplace violence.

### Descriptive Analysis of Study Variable

Table 2 shows scores of the 1,371 respondents' work stress, psychological job demands, and social approval. The mean score of ERR and over commitment were 1.2 ± 0.4 and 17.0 ± 2.7, respectively. 65.3% of the respondents experienced mismatch between high cost and low gain (ERR > 1). The mean score of psychological job demands was 10.8 ± 1.4, 0.6, 21.1 and 78.3% of the respondents had low, moderate and high psychological job demands, respectively. The mean score of social approval was 17.3 ± 3.2, 4.3% of the respondents experienced low social approval, 58.6 and 37.1% of the respondents experienced moderate and high social approval, respectively.

**Table 2:** Description of work stress, psychological job demands and social approval.

**Contents**	**Range**	**mean (SD)**
Work stress		
ERR	0.25–4	1.2 ± 0.4
Overcommitment	6–24	17.0 ± 2.7
Psychological job demands	3–12	10.8 ± 1.4
Social approval	5–25	17.3 ± 3.2

### Test of Study Model

**Figure 2:**
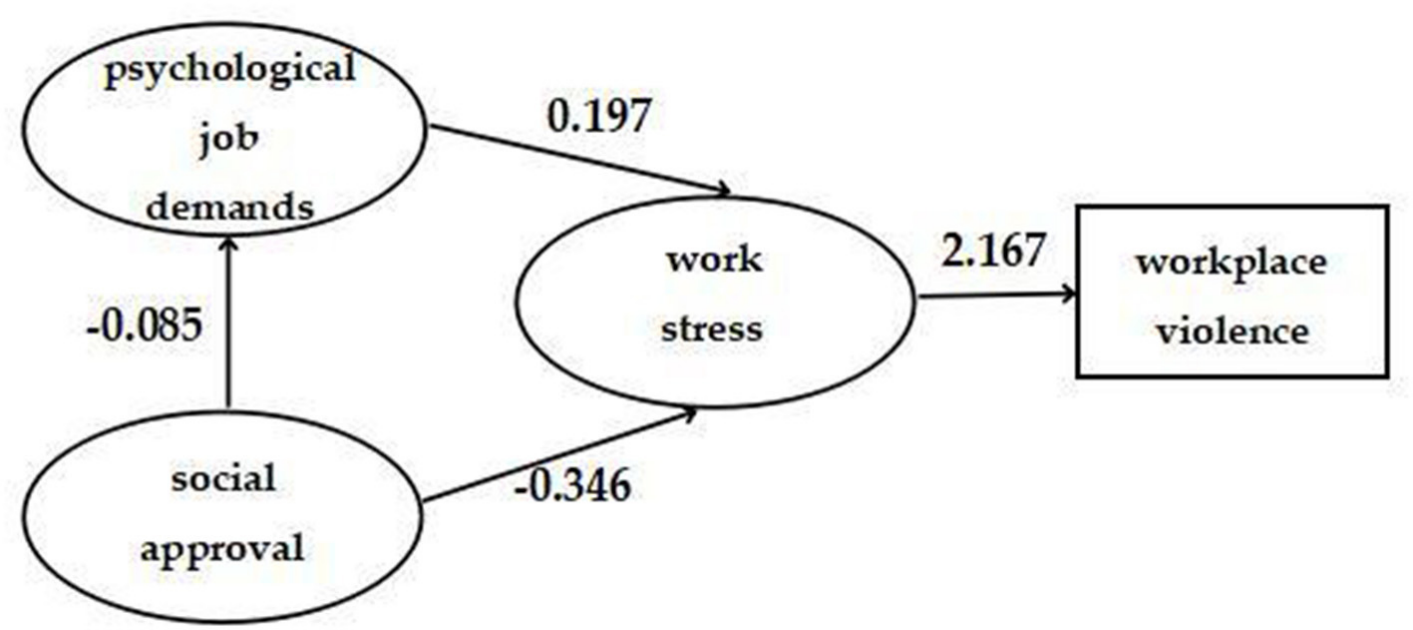
The final model and standardized model paths.

We used SEM to test the fitness of the hypothetical model in Figure 1. Figure 2 shows the final model where all paths were statistically significant and the model had an adequate fit: RMSEA = 0.047, TLI = 0.952, CFI = 0.972 and x^2^/df = 4.1.

Table 3 shows the results of hypothesis testing. As expected, work stress had a significant positive correlation with workplace violence (β = 2.167, 95%CI: 1.707, 2.627). Psychological job demands (β = 0.427, 95%CI: 0.297, 0.557) and social approval [β = −0.787, 95%CI: (−0.941)–(−0.633)] had only indirect associations with workplace violence, rather than direct associations. Psychological job demands had a direct association with work stress (β = 0.197, 95%CI: 0.139, 0.255). Social approval had direct association with work stress [β = −0.346, 95%CI: (−0.399)–(−0.294)] and psychological job demands [β = −0.085, 95%CI: (−0.136)–(−0.034)].

**Table 3:** Direct, indirect and total effects of key study variables.

**Pathways**	**Estimate**	**95%CI**
**Total effects**		
Work stress → Workplace violence	2.167	1.707, 2.627
Psychological job demands → Work stress	0.197	0.139, 0.255
Psychological job demands → Workplace violence	0.427	0.297, 0.557
Social approval → Work stress	−0.363	−0.418, −0.308
Social approval → Workplace violence	−0.787	−0.941, −0.633
Social approval → Psychological job demands	−0.085	−0.136, −0.034
**Direct effects**		
Work stress → Workplace violence	2.167	1.707, 2.627
Psychological job demands → Work stress	0.197	0.139, 0.255
Social approval → Work stress	−0.346	−0.399, −0.294
Social approval → Psychological job demands	−0.085	−0.136, −0.034
**Indirect effects**		
Psychological job demands → Workplace violence	0.427	0.297, 0.557
Social approval → Workplace violence	−0.787	−0.941, −0.633
Social approval → Work stress	−0.017	−0.027, −0.006

*CI, confidence interval*.

Table 4 shows the significance testing of the mediating pathways. In these analyses, if the 95% CI does not include 0, the mediating effect is statistically significant. The results indicated that the relationships of psychological job demands and social approval with workplace violence were both mediated by work stress (95% CI: 0.297, 0.557; −0.900, −0.601). In addition, psychological job demands mediated the relationship between social approval and work stress (95% CI: −0.027, −0.006).

**Table 4:** Significance test of mediating pathway.

**Pathways**	**95%CI**
Psychological job demands → Work Stress → Workplace violence	0.297, 0.557
Social approval → Work Stress → Workplace violence	−0.900, −0.601
Social approval → Psychological job demands → Work Stress	−0.027, −0.006

*CI, confidence interval*.

## Discussion

The study investigated the prevalence of workplace violence among health care workers in Sichuan province of China, and the purpose of this study was to explore the relationships among work stress, psychological job demands, social approval and workplace violence among health care workers, thereby providing theoretical support for future interventions for decreasing workplace violence among health care workers.

Our findings indicated that overall 77.0% of the health care workers reported the exposure to workplace violence in Sichuan province of China. It is higher than the results from previous studies in China [62.2% in 2015 (44), 68.6% in 2016 (45)]. One possible explanation may be that we included emotional abuse in our definition of workplace violence. In addition, this also indicates that the exposure rate of workplace violence among health care workers in China is generally high. According to Abdellah et al., about 75% of health care workers believe that workplace violence could be prevented (46). Thus, efforts should be strengthened to prevent health care workers from being exposed to workplace violence. In some studies, female health care workers are significantly more likely to be exposed to workplace violence (47, 48). But in this study, no statistically significant differences were found for sex which was consistent with previous research by Hahn et al. (49).

The mean score of overcommitment and ERR among health care workers were 17.0 ± 2.7, 1.2 ± 0.4, respectively, with more than half (65.3%) of the health care workers experiencing effort-reward imbalance. This is consistent with previous study conducted on Chinese health care workers by Cheng et al. (50). This suggests that the effort-rewards imbalance level is not low among health care workers in China. It may due to the fact that health care workers are often exposed to heavy workloads and was offered meager rewards compared to expected rewards. Previous survey that was conducted on Chinese health care workers reported that 12.7% experienced feelings of low self-accomplishment, 41.4% were dissatisfied with their salary, and 41.7% complained of a heavy workload (51). According to the Survey on the satisfaction of health care workers, most health care workers' dissatisfaction with the pay, workload evaluation and promotion opportunities to work were high (52). Thus, many Chinese health care workers face inadequate compensation economically and psychologically and experience an imbalance between the effort they make for their job and the rewards they receive.

The model supported that health care workers' work stress had a direct positive effect on workplace violence which is consistent with previous studies. Pekurinen et al. showed that work stress indicator (ERI) was associated with higher odds of patient aggression (53). Magnavita verified that the relationship between work stress and subsequent workplace violence remained significant even after adjusting for other confounding factors (12). One possible explanation may be that the health care workers who had high work stress and low rewards, are likely to elicit recurrent negative emotions. These negative emotions may result in poor commitment to aggression prevention practices through lowered work motivation, thus leading to the increased patients' dissatisfaction and higher odds of patient aggression (38). Alternatively, the social interactionist perspective has also suggested that stressed health care workers are likely to make more errors than their peers and are therefore perceived as less competent and targeted as victims of aggression (54). Therefore, to decrease workplace violence, efforts should be strengthened to decrease health care workers' work stress.

The results revealed that the psychological job demands of health care workers were high with a mean score of 10.8 ± 1.4. The model results supported that health care workers with higher psychological job demands were more likely to experience workplace violence which is consistent with previous studies (53). In the current study, we found that the relationship between psychological job demands and workplace violence was indirect rather than direct, with work stress functioning as the mediator. These findings are a meaningful addition to the existing literature and suggest that high psychological job demands cause health care workers to experience more work stress. One possible explanation may be that high psychological job demands lead to work stress for the health care workers, leading to interpersonal conflicts between two parties, which, when unresolved, will evolves into a bullying behavior (20). Thus, to prevent workplace violence, hospital managers should take organizational measures such as increasing staff, making adequate job design, and conducting administrative intervention to ease the psychological job demands to decrease work stress.

In this study, 58.6% of the health care workers experienced a moderate social approval with a mean score of 17.3 ± 3.2, indicating that the current practicing environment from the perspective of health care workers was not very good. The results are consistent with a previous national research of Chinese health professionals, which showed that the medical practice environment of health care workers was poor and was getting worse (55). One reason for the poor practice environment in China may be that media's reporting of adverse news negatively affected impression of health care workers in the public (56). Another possible explanation may be that the current medical resources can't match the public increasing requirements on the services capacity of medical institutions and health care workers, which leads to the public dissatisfaction and disrespect to health care workers, resulting in the deterioration in practice environment (55). Therefore, efforts should be strengthened to construct a supportive practice environment.

In another study on health care workers' experiences of workplace violence, limited social approval or practice environment has been linked to more workplace violence. Previous study showed nurses who work in poor practicing environments have greater odds of experiencing violence (32). However, the study was unable to clarify the mediating factors in this relationship. The current study showed that there was an indirect effect of social approval on workplace violence, but no direct effect. Adequate social approval appears to be associated with decreased psychological job demands and work stress, both of which were related to higher odds of workplace violence. Prior study has shown that practice environment factors are sources of work stress among health care workers (36, 37). Thus, health care workers with poor social approval from their practice environment may perceive higher psychological job demands and work stress. Consequently, they may experience more workplace violence.

Study limitations should be taken into account. First, despite the SEM being used to determine the relationship among the variables, the cross-sectional design imposes a significant limitation to drawing any definitive conclusions. In addition, we collected the data through the participants' self-report and submitted questionnaires rather than face-to-face investigation.

## Conclusions

The study shows that the exposure rate of workplace violence among health care workers in China is generally high. The results showed that work stress has a direct positive relationship with workplace violence, work stress also mediates the influence of psychological job demands and social approval on the workplace violence among health care workers. Therefore, priority should be given to interventions that target decreasing work stress. Paying more attention to increasing the public social approval to health care workers and decreasing the psychological job demands of the health care workers can decrease their work stress, thus decrease the exposure rate of workplace violence.

## Data Availability Statement

The original contributions presented in the study are included in the article/Supplementary Material, further inquiries can be directed to the corresponding author/s.

## Ethics Statement

The study protocol was approved by the Ethics Committee of the National Health Commission of the People's Republic of China. Verbal consent was obtained from each participant following a detail explanation about the purpose of the study. Written informed consent for participation was not required for this study in accordance with the national legislation and the institutional requirements.

## Author Contributions

XS and DL: conceptualization and methodology. XS: formal analysis and writing—original draft. XS, MQ, JZ, JP, XZ, and DL: investigation. MQ, JD, and DL: writing—review and editing. All authors contributed to the article and approved the submitted version.

## Funding

This research was funded by the Health Commission of Sichuan Province (Project identification code: H18 0933). The funders were not involved in the study design, analysis, interpretation of data, writing, or submission of this manuscript.

## Conflict of Interest

The authors declare that the research was conducted in the absence of any commercial or financial relationships that could be construed as a potential conflict of interest.

## Publisher's Note

All claims expressed in this article are solely those of the authors and do not necessarily represent those of their affiliated organizations, or those of the publisher, the editors and the reviewers. Any product that may be evaluated in this article, or claim that may be made by its manufacturer, is not guaranteed or endorsed by the publisher.
